# Micro-computed tomography of sandstone rocks: Raw, filtered and segmented datasets

**DOI:** 10.1016/j.dib.2022.107893

**Published:** 2022-02-02

**Authors:** Everton Lucas-Oliveira, Mariane Barsi-Andreeta, Rodrigo F. Neumann, Willian A. Trevizan, Mathias B. Steiner, Tito J. Bonagamba

**Affiliations:** a São Carlos Institute of Physics, University of São Paulo, PO Box 369, São Carlos, São Paulo 13560-970, Brazil; b IBM Research, Rio de Janeiro 20031-170, Brazil; c CENPES/Petrobras, Rio de Janeiro 21941-915, Brazil

**Keywords:** MicroCT, Rock core, Digital rock, Permeability, Porosity

## Abstract

High-resolution computed micro-tomography is an important area of science, which correlates well with several experimental methodologies and serves as a basis for advanced computational physics studies, in which high-resolution images are used as input to different scientific simulation models. The dataset presented herein includes (raw) grayscale images obtained using the Bruker Skyscan 1272 X-Ray tomograph; filtered images acquired through contrast enhancement and noise reduction filters; and segmented images obtained by using the IsoData segmentation method. All images have a resolution of 2.25 µm (isometric voxels) and size of 1000^3^ voxels.

## Specifications Table

**Table:** 

Subject	Earth and planetary sciences: Geophysics
Specific subject area	Porous Media characterization; High-resolution micro-computed tomography
Type of data	Images
How the data were acquired	X-Ray Microcomputed-tomography, Bruker Skyscan 1272
Data format	Raw, filtered and segmented image data (.raw)
Description of data collection	The data consist of a set of 3D images from 11 sandstone samples. Three images for each sample are provided: Raw Image, Filtered Image and Segmented Image. All images have a resolution of 2.25 µm (isometric voxels) and size of 1000^3^ voxels.
Data source location	Institution: Kocurek Industries, Hard Rock Division. City/Town/Region: Houston, TX.
Data accessibility	Repository name: Digital Rocks Portal Data identification number: 10.17612/f4h1-w124 Direct URL to data: https://dx.doi.org/10.17612/f4h1-w124
Related research article	Neumann, R.F., Barsi-Andreeta, M., Lucas-Oliveira, E. et al. High accuracy capillary network representation in digital rock reveals permeability scaling functions. *Sci Rep* 11, 11370 (2021). https://doi.org/10.1038/s41598–021–90090–0

## Value of the Data

• This dataset provides collection of 11 X-Ray microtomography 3D images of sandstone rocks spanning 10+ p.p. in porosity and 2 orders of magnitude in permeability.
• All the samples were measured under the same conditions, which is extremely rare for such a large dataset.
• Each image is offered in 3 variants (raw, filtered, and segmented), providing a ready-to-use image no matter what the use-case is.
• The development of computational methods for digital rock analysis, such as complex networks and artificial intelligence, can greatly benefit from the availability of a large, diverse, and standardized dataset like ours.
• Likewise, the development of new image processing methods, such as denoising, filtering, segmentation, and digital structural representation, can benefit from the same dataset.
• The dataset can be used by researchers in Digital Porous Media, Petroleum Science and Engineering, Water Science and Engineering, and Computational Fluid Dynamics who lack the resources to characterize these rock samples.
• More than 4000 users have already downloaded this dataset since its publication.

## 1 Data Description

The dataset [1] includes 3D microtomography images of sandstone samples acquired from Kocurek Industries: Bandera Gray, Parker, Kirby, Bandera Brown, Berea Sister Gray, Berea Upper Gray, Berea, Castlegate, Buff Berea, Leopard and Bentheimer. Researchers interested in using this dataset will find three different images for each sample:

­ *Raw image*: grayscale image data obtained from the reconstruction of the microCT projections.
­ *Filtered image*: grayscale image obtained from the original image through the application of contrast enhancement and noise reduction filters [2], [3].
­ *Segmented image*: binary image data obtained from the filtered grayscale image. The grayscale image was segmented at threshold level calculated using the IsoData algorithm [4].

All images have 1000 × 1000 × 1000 voxels, with isometric voxel size of 2.25 µm. It is worth mentioning that the segmented image includes isolated pores, unlike what is observed in the related work developed by Neumann [5] and Lucas-Oliveira [6], in which computational physics analysis were applied to systems where all pores can be saturated through pore connections.

## 2 Experimental Design, Materials and Methods

**Fig. 1:**
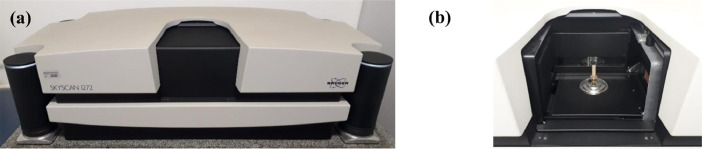
**(a)** Bruker Skyscan 1272 and **(b)** the cylindrical sample fixed on the *Rotational Step* of the Bruker Skyscan 1272.

The 3D images were acquired from cylindrical samples (height = 30 mm, radius = 5 mm) using a micro-computed tomography system Bruker Skyscan 1272, as shown in Fig. 1.

The acquisition parameters were configured as follows: Current of 100 µA; Voltage of 100 kV; Frame Averaging of 4; Cu 0.11 mm filter; Pixel Size of 2.25 µm; Rotation of 180° with 0.1° step.

**Fig. 2:**
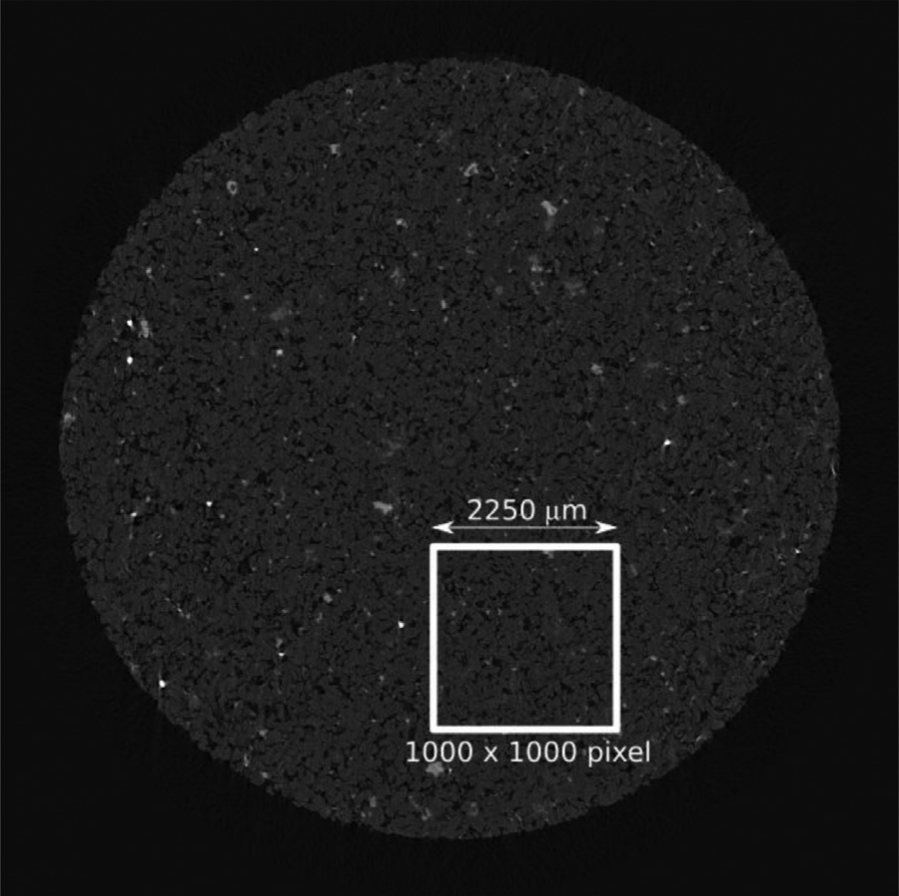
Example of micro-CT image with the selected area chosen for reconstruction, avoiding the center where the *Ring Artifact Correction* has more impact. An area of 1000 × 1000 pixels was selected within the sample region and 1000 slices were taken in the *z*-direction.

After the acquisition, the reconstruction was performed using the software provided by Bruker (NRecon, version 1.7.0.4, with the Reconstruction engine InstaRecon, version 2.0.2.6). To avoid the center of the image, where the *Ring Artifact Correction* has more impact, an area of 1000 × 1000 pixels was selected within the sample region, as in Fig. 2, with 1000 images in the *z* direction.

After image reconstruction, a contrast enhancement filter was applied. Different samples, with different mineralogic compositions, respond differently to X-ray transmission and yield a different distribution of grayscale levels. This step meant to equalize the contrast throughout, ensuring that all images were treated on equal footing by the subsequent image processing steps.

The contrast enhancement filter was applied to each image independently cutting off the grayscale histogram at the grayscale level in which the accumulated histogram achieved 99.8% and then mapping the remaining grayscale levels back to the [0, 255] interval. In this way, an efficient utilization of the gray level range was ensured.

The resulting images were later processed by a 3D non-local means filter [2], [3] implemented into the ImageJ software. The smoothing factor was chosen as 1 and the sigma parameter was determined automatically. This step reduces the amount of image noise that is inherent in any digital imaging technique.

**Fig. 3:**
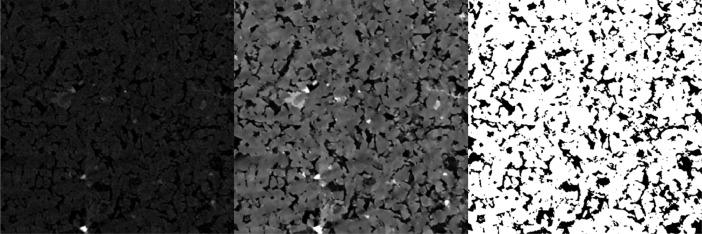
Example of raw (left), filtered (center) and segmented (right) Images, representing each step in our image processing workflow. Each image represents a square slice with 2250 µm of side.

Finally, the IsoData method [4] was used to compute a grayscale threshold level for each image, following their segmentation into solid and void spaces, leading to a binary image. In Fig. 3, all variants of a given image slice are shown side by side.

Both porosity and permeability were measured in commercial systems at the Petrobras Research Center (CENPES/Petrobras) and the properties were obtained for cylindrical sandstone samples (diameter and length of 38 mm). Porosity and absolute permeability were measured at an overburden pressure of 500 psi in UltraPore 300 and UltraPerm 500 equipment (both from Core Lab, USA), respectively, using Nitrogen gas at 25 °C. Both permeability and porosity are published in reference [5].

## CRediT authorship contribution statement

**Everton Lucas-Oliveira:** Conceptualization, Investigation, Validation, Methodology, Formal analysis, Writing – original draft. **Mariane Barsi-Andreeta:** Conceptualization, Methodology, Software, Formal analysis, Writing – review & editing. **Rodrigo F. Neumann:** Conceptualization, Methodology, Software, Formal analysis, Writing – original draft. **Willian A. Trevizan:** Resources, Writing – review & editing. **Mathias B. Steiner:** Supervision, Conceptualization, Writing – original draft, Writing – review & editing. **Tito J. Bonagamba:** Supervision, Conceptualization, Writing – original draft, Writing – review & editing.

## Declaration of Competing Interest

The authors declare that they have no known competing financial interests or personal relationships that could have appeared to influence the work reported in this paper.
